# Hepatic S1P deficiency lowers plasma cholesterol levels in apoB-containing lipoproteins when LDLR function is compromised

**DOI:** 10.1186/s12986-015-0031-4

**Published:** 2015-10-20

**Authors:** Debapriya Basu, Afroza Huq, Jahangir Iqbal, M. Mahmood Hussain, Xian-Cheng Jiang, Weijun Jin

**Affiliations:** Department of Cell Biology, State University of New York, Downstate Medical Center, Brooklyn, NY 11203 USA; Department of Pediatrics, State University of New York, Downstate Medical Center, Brooklyn, NY 11203 USA

**Keywords:** S1P, Knockdown, cre, Blp-c, *Ad libitum*, LDLR, PCSK9, SREBP, Hepatocytes

## Abstract

**Background:**

Site-1 protease (S1P) is the key enzyme required for activation of the sterol regulatory element binding proteins (SREBPs) that govern lipid synthesis. While S1P has been speculated to influence plasma apoB-containing lipoprotein (Blp) metabolism, there has been little investigative work. LDL receptor (LDLR) is the major receptor for clearing plasma LDL cholesterol (LDL-c). Proprotein convertase subtilisin kexin type 9 (PCSK9) modulates LDL-c through post-translational degradation of the LDLR.

**Methods:**

A hepatic-specific knockdown (KD) of S1P was achieved using floxed S1P mouse models (S1P^f/f^ and LDLR^-/-^S1P^f/f^) and hepatic expression of Cre recombinase. Lipids were measured in total plasma and size fractionated plasma using colorimetric assays. Realtime polymerase chain reaction, western blotting and ELISA were used to determine hepatic expression of key genes/protein. Plasmid mediated overexpression and siRNA mediated knockdown of genes were performed in mouse primary hepatocytes to determine the mechanistic basis of PCSK9 gene regulation.

**Results:**

A hepatic-specific KD of S1P resulted in a 45 % and 38 % reduction in plasma total cholesterol and triglyceride levels, respectively. Hepatic S1P KD had a minimal effect on plasma Blp cholesterol (Blp-c) in S1P^f/f^ mice, despite significantly reducing VLDL secretion. Notably, hepatic S1P KD decreased the LDL receptor (LDLR) mRNA expression by 50 %. However, the reduction in LDLR protein levels was less than that of mRNA expression, especially under fed conditions. Further assessment of hepatic S1P deficiency revealed that it increased LDLR protein stability *in vivo*. Mechanistically, hepatic S1P KD was shown to decrease the liver and plasma levels of the protein proprotein convertase subtilisin/kexin type 9 (PCSK9), which degrades LDLR protein. This effect was more prominent in the fed condition and sufficient to account for the discordance in LDLR mRNA and protein levels. Furthermore, hepatic S1P was shown to regulate PCSK9 expression through activation of the SREBPs. In the LDLR^-/-^ background, hepatic S1P KD significantly reduced Blp-c levels.

**Conclusion:**

Hepatic S1P is a physiological modulator of plasma Blp metabolism through its regulation of LDLR and PCSK9. Hepatic S1P is a valid target for lowering plasma Blp-c levels in the situation where LDLR function is compromised.

## Background

Site 1 protease (S1P, also known as membrane-bound transcription factor peptidase, site 1), belongs to the proprotein convertase (PCSK) family. The S1P precursor undergoes an auto-catalytic processing in the ER, involving sequential cleavage at two sites, to generate two active mature forms, B and C [1]. The mature S1P forms (henceforth referred to as S1P) then become anchored to the *Golgi* membrane [1]. S1P cleaves and activates membrane-bound unprocessed transcription factors that have been transported to the *Golgi* [2–5]. For example, S1P is one of the key enzymes required for release of the transcription-stimulating domains of sterol regulatory element binding proteins (SREBP1a, SREBP1c and SREBP2) *in vitro* and *in vivo* [6]. SREBPs are synthesized as inactive precursors bound to endoplasmic reticulum (ER) in complex with SCAP, an ER to Golgi transport protein.

SREBPs are the best characterized of the transcription factors which regulate *de novo* synthesis of fatty acids and cholesterol. Although SREBP1 and 2 bind to the same consensus sequence, for unknown reasons SREBP1c chiefly regulates the synthesis of fatty acids by enhancing transcription of the genes encoding acetyl CoA carboxylase, fatty acid synthase, stearoyl CoA desaturase-1 and other genes related to fatty acid synthesis [7]. SREBP2, on the other hand, controls the synthesis of cholesterol by increasing the levels of mRNAs for all known enzymes of the cholesterol biosynthetic pathway [7]. When S1P is inactivated or inhibited [6, 8], levels of the active forms of the SREBPs in hepatocytes fall markedly, along with a decline in all SREBP target mRNAs, and the rates of synthesis of fatty acids and cholesterol decline by approximately 90 % and 75 %, respectively [6, 8].

Interestingly, S1P has been shown to influence plasma lipid levels in three different mouse models fed a chow diet: (i) a hypomorphic S1P mutation reduced total cholesterol (TC) levels by 50 % but not triglyceride levels (TG) [9]; (ii) acute disruption of S1P in hepatocytes and cells of myeloid lineage, mediated by an inducible Cre recombinase, led to a decline in plasma TC and TG levels [6]; (iii) pharmacological inhibition of S1P significantly decreased TC and TG concentrations (8). While it has been speculated that S1P affects plasma apoB-containing lipoprotein (Blp) metabolism, there has been little investigative work. This is of particular importance since lowering plasma Blp-c levels is the cornerstone of current management of cardiovascular disease.

The mechanism(s) by which liver S1P regulates plasma lipid metabolism is (are) not fully understood. Since hepatic S1P inhibition decreases the activation of SREBPs and hence reduces *de novo* lipid synthesis, the reduced lipid substrates should reduce VLDL assembly and lead to lower VLDL secretion rates. One would expect an associated decrease in the plasma cholesterol levels in Blps, including VLDL, IDL and LDL. However, activation of the SREBP pathway by constitutively expressing the SREBP transcription stimulating domains also lowers plasma TC and TG levels [10–12] , suggesting that the reduced plasma lipid levels following S1P deficiency/inhibition cannot be exclusively explained by changes in *de novo* lipid synthesis.

Potential mechanisms are many, since many genes in addition to those involved in fatty acid and cholesterol biosynthetic pathways are regulated by the SREBPs [13], including those for the LDL receptor (LDLR) and proprotein convertase subtilisin/kexin type 9 (PCSK9). S1P is also the candidate protease for activation of two other major transcription factors involved in lipid metabolism, activating transcription factor 6 (ATF6) [3], and cyclic AMP-responsive element binding protein-H (CREBH) [4]. S1P could thus affect plasma lipid metabolism by pathways in addition to those involving the SREBPs.

The major questions underlying this research are: does inhibition of hepatic S1P decrease plasma Blp-c levels? If so, what is the molecular mechanism? We evaluated the role of hepatic S1P in plasma Blp-c metabolism using different mouse models of hepatic S1P deficiency. We showed that hepatic S1P inhibition lowers plasma Blp-c levels in LDLR^-/-^S1P^f/f^ but not *S1P*^*f/f*^ mice. We found that hepatic S1P, by controlling the activation of SREBPs, is a physiological modulator of liver LDLR and PCSK9. These two regulations are critical for fine-tuning LDLR function and hence plasma Blp-c levels.

## Methods

### Materials

All reagents and chemicals were obtained from Fisher Scientific (Pittsburgh, PA) and Sigma Aldrich (St. Louis, MO) unless otherwise stated.

### Animals

All procedures were conducted in conformity with the United States Public Health Service Policy on Humane Care and Use of Laboratory Animals and approved by the Institutional Animal Care and Use Committee of SUNY Downstate Medical Center. All mice used have a C57Bl/6 J background. S1P heterozygous floxed mice were obtained from The Jackson Laboratory (Bar Harbor, Maine) [6] and bred to generate S1P homozygous floxed mice (S1P^f/f^). Mice were studied at 8-12 weeks of age. Studies were carried in both male and female mice and similar results were obtained. To achieve acute knockdown (KD) of S1P in the adult mouse liver, S1P^f/f^ mice were intravenously administered adenovirus-expressing Cre under the CMV promoter (S1P^f/f^Cre mice). They were studied 4–7 days after virus administration. Control group S1P^f/f^ mice were intravenously administered adenovirus expressing either firefly luciferase (S1P^f/f^Luci mice) or adenovirus not expressing any protein (S1P^f/f^Empty mice). 1 × 10^11^ viral particles were injected per mouse. Albumin Cre mice were purchased from The Jackson Laboratory [14]. S1P^f/f^ mice were crossed with these mice to obtain mice with liver-specific ablation of S1P (L-S1P). S1P^f/f^ mice were bred with LDLR knockout (LDLR^-/-^) mice to obtain LDLR^-/-^S1P^f/f^ mice. Primer sequences for genotyping were obtained from The Jackson Laboratory.

Mice were kept on a 7 AM-7 PM lighting schedule with free access to water and a standard laboratory chow diet unless otherwise indicated. *Ad libitum* fed mice were sacrificed between 9 AM-10 AM. For overnight fasting studies, the chow diet was withdrawn from 6 PM-9 AM. For collection of plasma, mice were anesthetized using isoflurane and blood was collected from the retro-orbital plexus in heparinized micro-capillary tubes, centrifuged at 10,000 × g at 4 °C, used immediately and/or stored at −20 °C. For organ collection, anesthetized mice were perfused with PBS to remove blood and then livers and other tissues were collected and snap frozen in liquid nitrogen or placed in dry ice and then stored at −80 °C.

### RNA isolation, cDNA synthesis and real-time PCR

RNA was isolated from livers or cells using the GeneJet RNA isolation kit (Thermo Scientific, Waltham, MA). RNA concentration was measured using the Nanodrop 2000 (Thermo Scientific, Waltham, MA) and 1.5 μg RNA per sample was used for cDNA synthesis using the Verso cDNA kit (Thermo Scientific, Waltham, MA). cDNA was amplified with gene specific primers and detected with the Absolute Blue SYBR Green ROX Mix (Thermo Scientific, Waltham, MA) in the StepOne Plus Realtime PCR machine (Applied Biosystems, Grand Island, NY). 18S RNA was used as an invariant control. The standard curve method of analysis for RNA quantitation was utilized using dilutions of corresponding cDNA plasmids as the standards. The primer sequences for S1P, PCSK9, LDLR, SREBP1c, SREBP2, ACC1, FAS and HMGCoA reductase were as described in a previous publication [6]. All other primer sequences for real-time PCR were obtained from the Primer Bank (http://pga.mgh.harvard.edu/primerbank/).

### Western blot analysis

Liver tissue from mice was homogenized in RIPA buffer supplemented with Protease Inhibitor Cocktail from Sigma (St. Louis, MO) using a QIA Tissue Lyser (Qiagen, Valencia, CA). The homogenates were centrifuged at 14,000 rpm for 15 min at 4 °C and the supernatants collected. Protein concentration was measured using a BCA kit from Pierce (Rockford, IL). Supernatant (50 μg protein) was combined with 4 × concentrated SDS loading buffer and reducing agent and heated at 80 °C for 10 min. The proteins were separated on 4–20 % SDS PAGE gels from Pierce (Rockford, IL), transferred electrophoretically to nitrocellulose membranes, blocked with 5 % bovine serum albumin and probed with the following primary antibodies (Abs): anti-LDLR from R&D Biosciences (Minneapolis, MN); anti-GAPDH from Epitope Biotech Inc (Burnaby, B.C., Canada); anti-S1P from Santa Cruz Biotech Inc (Santa Cruz, CA); anti-actin from Genscript (Piscataway, NJ). Horse radish peroxidase conjugated secondary Abs were obtained from Jackson Immunoresearch (West Grove, PA) and the visualized using the ECL Super Signal West Pico or Femto reagent from Pierce (Rockford, IL). Bands were scanned using a Microtek ScanMaker 5950 (Microtek, Santa Fe Springs, CA) and quantified using Image-Pro Plus 6.0 (Media Cybernetics, Warrendale, PA).

### Lipid analysis

Total cholesterol and triglyceride were determined using commercially available kits from Fisher Scientific (Pittsburgh, PA). Lipoprotein profiles in pooled plasma samples were obtained by fast-protein liquid chromatography (FPLC) using a gel filtration column as described [15, 16], each sample comprising equal amounts of plasma from each of the mice constituting an experimental group.

### VLDL-TG production *in vivo*

Four-hour fasted mice were injected intraperitoneally with poloxamer 407 (P407) dissolved in PBS. P407 inhibits lipoprotein lipase, endothelial lipase and hepatic lipase activities, preventing the degradation of plasma TG-rich lipoproteins, which build up in the circulation. Blood was collected before (0 h) and at 1, 2, 4 and 6 h post P407 injection and plasma TG were measured.

### Plasma PCSK9 measurements

The Circulex Mouse PCSK9 ELISA kit from MBLI (Woburn, MA) and the Mouse PCSK9 Quantikine kit from R&D Biosystems (Minneapolis, MN) were used according to the manufacturers’ instructions.

### Hepatocyte isolation and culture

S1P^f/f^ mice (transduced with either Ad-Luciferase or Ad-Cre) were anesthetized with an intraperitoneal injection of Ketamine (100 mg/kg) and Xylazine (10 mg/kg). Each mouse was perfused with Hank’s balanced salt solution (buffered with Hepes to a final concentration of 10 mM, pH 7.4) for 5 min through the inferior vena cava using a MP II Mini-Peristaltic Pump (Harvard Apparatus, Holliston, MA) at a setting of 6 ml/min until the liver was free of blood. The liver was then perfused with collagenase type I (Worthington Biochemicals, Lakewood, NJ) added at 0.4 mg/ml to 1 × Hank’s solution and incubated for 15 min. The collagenase digested liver was transferred into 25 ml of ice-cold William’s Medium E from Sigma Aldrich (St. Louis, MO). The liver capsule was cut open, hepatocytes were dispersed into the media with blunt forceps, filtered through a 70 μm filter ( BD Bioscience, Mississauga, ON) and combined with 25 ml of Percoll solution (2.5 ml of 10 × Hank’s solution plus 22.5 ml of Percoll). The cell suspension was centrifuged at 180 × g for 6 min to obtain live cells as a pellet. The pellet was washed with 25 ml of William’s Medium E solution to remove traces of Percoll. The washed hepatocytes were then resuspended in DMEM containing 10 % FBS. Cell viability as checked by Trypan Blue exclusion was approximately 90 %. Hepatocytes were then plated in collagen-coated plates (BD Bioscience, San Jose, CA). After 2 h incubation at 37 °C with 5 % CO_2_ when live cells had attached, the culture media was changed and fresh DMEM with10% FBS added [15].

### siRNA transfection

siGenome Smartpool siRNAs were obtained from Dharmacon (Pittsburgh, PA) and transfected into primary hepatocytes at 40 nM final concentration using the Lipofectamine 2000 transfection reagent from Invitrogen (Grand Island, NY) [17]. Non-targeting scrambled siRNAs were used as controls and were denoted as siScr. Hepatocytes were collected for RNA isolation 56 h post transfection.

### Luciferase constructs and assays

The PCSK9 promoter regions (1 kb and 3 kb starting at the transcription site) [18] were amplified from mouse liver genomic DNA and cloned into a Gaussia luciferase reporter construct (New England Biolabs (NEB), Ipswitch, MA). All plasmid constructs were verified by DNA sequencing prior to transfection in cells. The Cypridina luciferase construct from NEB was used as the control plasmid for *in vitro* dual reporter assays. Gaussia and Cypridina luciferases are secreted into the cell culture media. Plasmids were transfected into primary hepatocytes with Lipofectamine 2000 [19]. For dual luciferase measurements, cell culture media were collected 48 h post transfection. For most studies, 10 μl of media was used to measure Gaussia and Cypridina activities and the ratio of Gaussia/Cypridina activities was calculated as an indicator of PCSK9 promoter activity.

### DiI-LDL binding and uptake

Fluorescent DiI-LDL, Biomedical Technologies, Inc. (Stoughton, MA) was diluted in serum free DMEM (5ug/ml), added to hepatocytes and incubated at 4 °C for 2 h for binding and at 37 °C for 3 h for uptake [20]. The media was then removed, cells were washed gently with PBS and then lysed in RIPA buffer. The lysate was centrifuged at 12,000 rpm for 5 min and the fluorescence in the supernatant quantified using a Spectramax M2 plate reader. The values were normalized to the protein concentration of the cell lysates.

### Nuclear run-on assay

Intact nuclei were isolated from murine primary hepatocytes and an *in vitro* nuclear run-on assay was carried out using biotinylated UTP from Roche (Indianapolis, IN). Total RNA was isolated and the newly synthesized RNA which was biotinylated was pulled down using streptavidin magnetic beads Pierce (Rockford, IL). Beads were washed rigorously and then used for cDNA synthesis. Real-time PCR was used to quantify cDNA from newly synthesized PCSK9 pre-mRNA and 18S mRNA [21].

## Statistical analyses

Values are expressed as the mean ± standard deviation (SD) of triplicate determinations. Mice experiments were repeated three times or more to confirm the consistency of results. Comparisons between two groups were analyzed by the unpaired Student’s *t* test. Comparisons among multiple mouse groups were analyzed by ANOVA followed by unpaired Student’s *t* tests if necessary to determine significant differences.

## Results

### Generation of hepatic S1P KD models

**Fig. 1:**
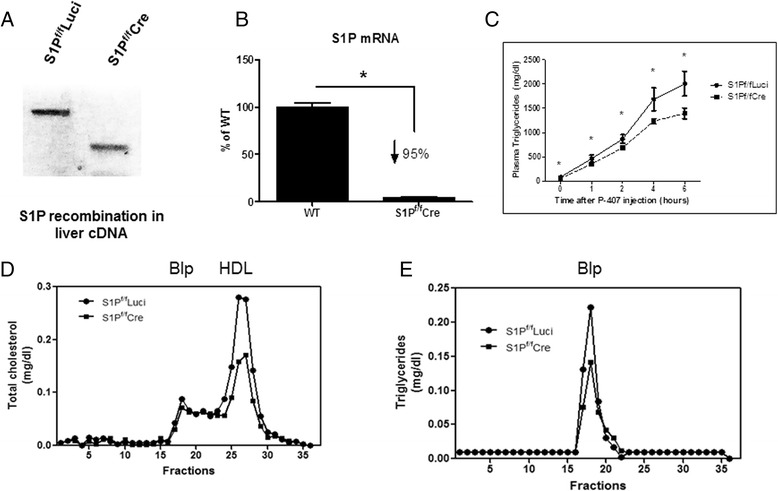
Hepatic S1P KD and plasma lipid levels. Hepatic S1P KD was verified by (**a**), Reverse transcriptional PCR analysis of liver cDNA using primers that binds to sequence outside of the floxed sites; (**b**), Real-time PCR analysis of S1P KD in hepatocytes from S1P^f/f^Cre and WT control mice. *N* = 4, each data point represents the mean +/- S.D., * denotes *p* < 0.05; (**c**), VLDL-TG secretion study. Four-hour fasted female S1P^f/f^ Luci and S1P^f/f^Cre mice were injected intraperitoneally with poloxamer 407 (P407) and plasma TGs monitored over a period of 6 h. *N* = 4, 6–8 weeks of age each data point represents the mean +/- S.D., * denotes *p* < 0.05; (**d**-**e**), Lipid measurements were carried out on gel filtration fractions from the pooled plasma samples. This experiment was repeated three times and similar results were obtained

S1P^f/f^ mice described by Yang et al. [6] have about 20 % of the S1P mRNA levels of wild-type (WT) mice likely due to the *neo* cassette interfering with transcription of S1P. However S1P^f/f^ mice are indistinguishable from WT mice in all other respects including plasma lipid profile [6]. To determine the physiological role of hepatic S1P in regulating plasma lipid and lipoprotein levels, we have used S1P^f/f^ mice and knocked down S1P using two well-accepted approaches: (a), S1P^f/f^ Cre, a model of acute hepatic S1P knockdown (KD) via adenovirus-mediated Cre expression, and (b), L-S1P mice (S1P^f/f^ crossed with Albumin-Cre), a model of liver-specific S1P KD via a Cre transgene under the control of the albumin promoter. As expected, the *S1P* gene, when subjected to Cre-induced recombination, produced a shorter nonfunctional transcript (Fig. 1a). Levels of the full-length hepatic S1P transcript were reduced compared with those of S1P^f/f^ controls (Table 1, columns 1 and 2). The reduction in S1P mRNA expression was further confirmed in freshly isolated primary hepatocytes (Fig. 1b).

**Table 1:** Phenotypic comparison of S1P^f/f^, and LDLR^-/-^S1P^f/f^ after hepatic S1P KD

Parameter	S1P^f/f^		LDLR^-/-^S1P^f/f^	
	Ad-Luci	Ad-Cre	Ad-Luci	Ad-Cre
Number and sex	4 females	4 females	4 females	4 females
S1P mRNA %^a^	100 ± 7.8	23.3 ± 3.0**	100 ± 3.8	29 ± 15.4**
TC, mg/dl	78.5 ± 13.2	43.7 ± 7.0*	180.8 ± 31.9	109.9 ± 14.4*
TG, mg/dl	111.2 ± 20.5	69.5 ± 7.0*	107.3 ± 36.9	73.8 ± 16.6*

Female mice (6–8 weeks of age) were injected i.v. with either Ad-Luci or Ad-Cre as described in Methods. Eight days after the last injection, blood was obtained. Each value represents the mean ± the SD of four mice. The statistical significance (Student’s *t* test) between Ad-Luci and corresponding Ad-Cre mice was calculated. *P < 0.05; and **P < 0.01. ^a^full length S1P mRNA expression is expressed as a % of the corresponding Ad-Luci value

### Hepatic S1P KD lowered plasma TC and TG but showed minimal effect on plasma Blp-c levels

S1P^f/f^ Cre and L-S1P mice have similar knockdown of S1P in the liver and gave similar results with respect to effects on plasma lipid phenotypes (Table 1, columns 1 and 2; Table 2). We have focused on the S1P^f/f^ Cre mice model as it eliminates the gene in the adult mice and mimics drug intervention. In contrast, albumin cre mice express cre right after birth.

**Table 2:** Phenotypic comparison of S1P^f/f^ and L-S1P

Parameter	S1P^f/f^	L-S1P
Number and sex	5 males	5 males
S1P mRNA %^a^	100 ± 39.2	15 ± 8.4**
TC, mg/dl	68.8 ± 6.3	49.7 ± 5.3*
TG, mg/dl	52.7 ± 10.7	36.9 ± 7.5*

Blood was obtained from non-fasted male mice (6–8 weeks of age) as described in Methods. Each value represents the mean ± the SD of five mice. The statistical significance (Student’s *t* test) between S1P^f/f^ and L-S1P was calculated. *P < 0.05; and **P < 0.01. ^a^full length S1P mRNA expression is expressed as a % of the corresponding S1P^f/f^ value

The physiological effect of hepatic S1P KD on plasma lipid levels was investigated following *ad libitum* feeding. In the S1P^f/f^ Cre mice, 45 % and 38 % reductions in plasma TC and TG levels, respectively, were observed compared with S1P^f/f^Luci controls (Table 1, columns 1 and 2). These results are in agreement with published work indicating that hepatic S1P is a major regulator of plasma lipids [6].

S1P KD in hepatocytes has been shown to reduce fatty acids and cholesterol secretion *in vitro* [6]. We hypothesized that hepatic S1P would reduce VLDL secretion *in vivo*. VLDL secretion was studied in S1P^f/f^ Cre and S1P^f/f^ control mice. Four-hour fasted mice were injected intraperitoneally with P407 and blood was collected over a period of 6 h. P407 inhibits lipoprotein lipase, endothelial lipase and hepatic lipase activities, preventing the degradation of plasma TG-rich lipoproteins [11]. Since, VLDL particles are mainly TG rich, we measured plasma TG levels at different time points. As shown in Fig. 1c, significantly less TG was secreted in S1P^f/f^ Cre than in control animals. This *in vivo* experiment showed that hepatic S1P modulates VLDL secretion and hence plasma lipids.

To determine which lipoprotein fractions are affected by hepatic S1P KD, pooled plasma samples from the fasted mice in each group were fractionated by FPLC and cholesterol and TG concentrations were measured in different fractions. Hepatic S1P KD produced similar relative decreases in both HDL-c (Fig. 1d) and Blp TGs (Fig. 1e). However, hepatic S1P KD had a minimal effect on plasma Blp cholesterol (Blp-c) (Fig. 1d), despite the significant reduction it produced in VLDL secretion (Fig. 1c). Similar results were obtained when the experiment was repeated on three other occasions.

### Hepatic S1P deficiency resulted in discordance between LDLR mRNA and protein levels in mice fed ad libitum

**Fig. 2:**
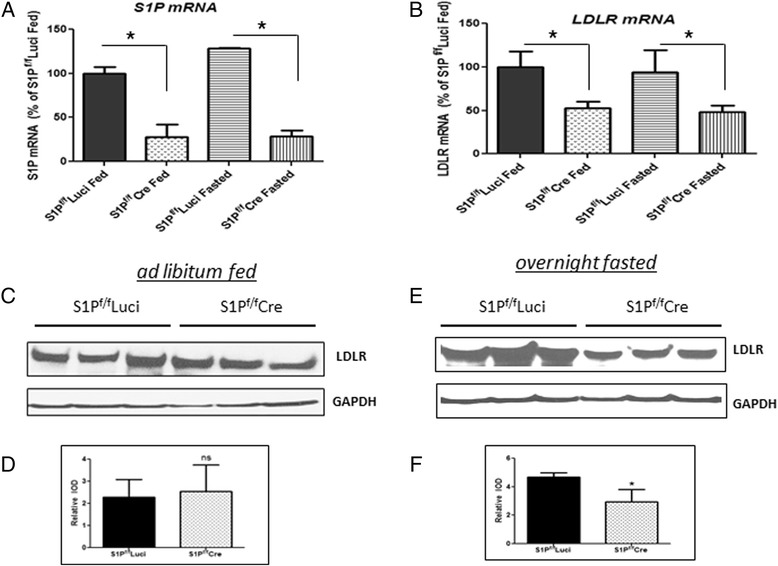
Comparison of mRNA and protein levels of the LDLR in the livers of S1P^f/f^ Luci and S1P^f/f^Cre mice *ad libitum* fed or fasted as described in the Methods Section. 6–8 weeks female mice were sacrificed at 9 AM. **a**, Hepatic S1P mRNA; **b**, LDLR mRNA; **c** and **d**, LDLR protein levels; **e** and **f**, quantification of the Western blots. *N* = 4, each data point is the mean +/- SD, *, denotes *p* < 0.05. ns, denotes not significant

To investigate why hepatic S1P KD had little effect on plasma Blp-c levels, we first evaluated hepatic LDLR expression which is a major regulator of LDL-c. S1P^f/f^Luci and S1P^f/f^Cre mice in the *ad libitum* fed state (Fig. 2). Hepatic S1P KD (Fig. 2a, fed state) reduced hepatic LDLR mRNA by 50 % (Fig. 2b, fed state). This is in agreement with earlier studies using mice in the fed state [6, 8]. Despite this reduction in LDLR mRNA, liver LDLR protein levels in *ad libitum* fed S1P^f/f^Cre mice were similar to those of S1P^f/f^Luci control mice (Fig. 2c and d, fed state).

When S1P^f/f^Luci and S1P^f/f^Cre mice were fasted overnight, levels of hepatic LDLR mRNA in S1P^f/f^Cre mice were reduced compared with control mice (Fig. 2b, fasted state) to an extent similar to that found in *ad libitum* feeding conditions (Fig. 2b, fed state). However, LDLR protein levels in the fasted state in S1P^f/f^Cre mice were significantly lower than that of the control mice (Fig. 2e and f). Similar results were obtained when mice were fasted for 4 h (data not shown). Therefore, hepatic S1P KD results in reduced LDLR function by decreasing LDLR mRNA transcription, which in the fasting state leads to reduced LDLR protein expression. The above results provide an explanation for why hepatic S1P KD has a minimal effect on plasma Blp-c levels in S1P^f/f^Cre mice fasted for four hours (Fig. 1d). Hepatic S1P KD decreases VLDL secretion (Fig. 1c) in fasted mice, which leads one to expect a reduction in plasma Blp-c levels. However, hepatic S1P KD reduces LDLR protein expression in these mice (Fig. 2e), which would impair uptake of Blp particles and thus normalize Blp-c levels.

### Hepatic S1P deficiency regulates LDLR protein levels post-translationally

**Fig. 3:**
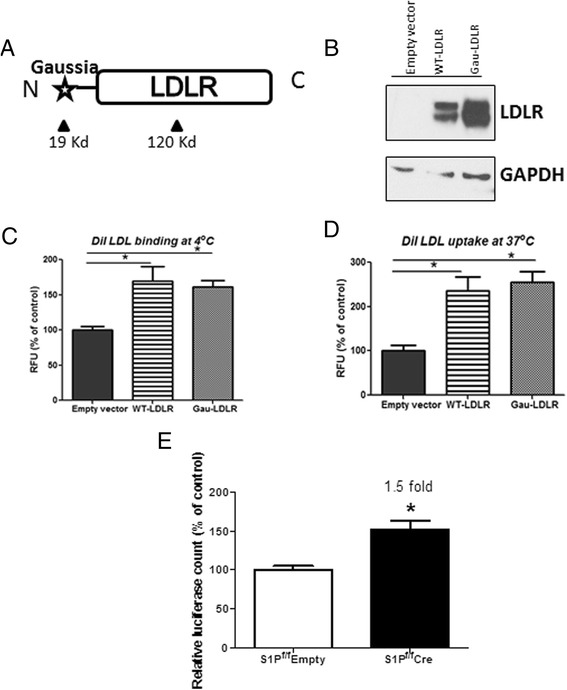
LDLR protein is stabilized in S1P^f/f^ Cre mice livers. **a**, A schematic depicting the Gau-LDLR fusion protein reporter; **b**, Huh7 cells were transfected with empty vector, wild-type LDLR or a Gau-LDLR plasmid. Forty-eight hours after transfection, cell lysates were collected and LDLR expression was confirmed by immunoblotting; **c** and **d**, Another two sets of cells were subjected to DiI-LDL binding or uptake, respectively, as described in Materials and Methods; **e**, Relative luciferase (Gaussia/Firefly) counts in livers of S1P^f/f^ Empty and S1P^f/f^Cre mice as a measure of total LDLR protein as described in Material and Methods. Here Ad-Empty was used for S1P control mice as Ad-luciferase was administered in both control and S1P KD mice to serve as the normalizing factor for injection. *N* = 3, each data point is mean +/- SD, * denotes *p* < 0.05

After observing the discordance between LDLR mRNA and protein levels in *ad libitum* fed S1P deficient mice, we hypothesized that hepatic S1P also regulates LDLR protein levels at the post-translational level. We generated a plasmid expressing the murine LDLR with an added N-terminal Gaussia luciferase, designated Gau-LDLR, controlled by the CMV promoter (Fig. 3a). The resultant fusion protein was used to monitor the turnover of endogenous LDLR protein. Expression of the Gau-LDLR fusion protein was verified in Huh7 cells using immunoblotting (Fig. 3b). *In vitro* DiI-labeled LDL binding and uptake assays confirmed that the Gau-LDLR protein was functional (Fig. 3c and d).

To assess post-translational regulation of the LDLR *in vivo*, we created a dual reporter assay by transducing mice with Ad-Gau-LDLR in combination with Ad-Firefly luciferase (Ad-Luci): the latter to serve as a control for transduction efficiency. The assay was carried out in liver homogenates from S1P^f/f^Empty and S1P^f/f^Cre mice fed *ad libitum*. A higher ratio of Gaussia/Firefly luciferase activities was obtained in the supernatants of S1P^f/f^Cre liver homogenates compared with those from S1P^f/f^Empty, indicating a greater total LDLR protein stability in S1P deficient mice livers (Fig. 3e).

### Hepatic S1P KD decreases PCSK9 expression

**Fig. 4:**
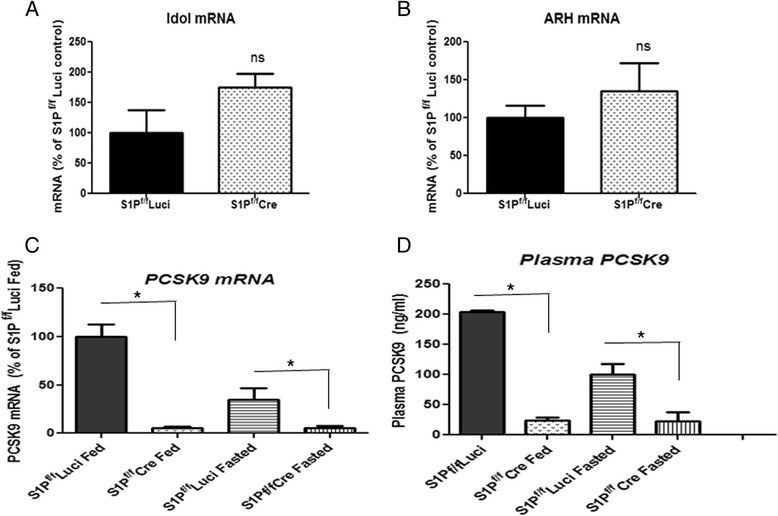
Hepatic S1P KD and regulators of the LDLR. **a** and **b**: Hepatic mRNA analyses in S1P^f/f^Luci and S1P^f/f^Cre mice using real-time PCR. **a**, Idol; **b**, ARH; **c** and **d**: studies in the mouse models of Fig. 2: **c**, PCSK9 mRNA levels; **d**, Plasma PCSK9 protein levels quantified using ELISA. *N* = 4, each data point represents the mean +/- SD * denotes *p* < 0.05. ns denotes not significant

To investigate how S1P KD enhances hepatic LDLR protein stability, we measured hepatic mRNA levels of several post-translational regulators of LDLR protein in the presence and absence of S1P KD. Inducible degrader of the LDLR (IDOL) [20] and Autosomal Recessive Hypercholesterolemia (ARH, [22]) mRNA levels were not altered when S1P was knocked down (Fig. 4a and b).

Since PCSK9 protein degrades the LDLR [23, 24], we determined the effect of hepatic S1P KD on PCSK9 expression in mice in two conditions: following *ad libitum* feeding and after overnight fasting. S1P deficiency reduced the levels of liver PCSK9 mRNA and plasma PCSK9 protein in S1P^f/f^Cre compared with S1P^f/f^Luci animals in both conditions (Fig. 4c and d). However, the reduction was greater after *ad libitum* feeding than after fasting (PCSK9 mRNA: 13- versus 5-fold reduction, *p* < 0.05; PCSK9 protein: 8.3- versus 4.5-fold reduction, *p* < 0.05). These results indicate that hepatic S1P KD more significantly decreases PCSK9 expression in the fed state. This would reduce PCSK9-mediated LDLR protein degradation in the S1P KD mice livers and normalize LDLR protein to that of control mice in the fed state than in fasting conditions. Thus, the observed greater reduction in plasma PCSK9 protein after hepatic S1P KD in the fed state provides an explanation for the discordance between LDLR mRNA and protein expression in the fed state (Fig. 2b, c  and d).

### Hepatic S1P KD decreases PCSK9 expression at the transcriptional level

**Fig. 5:**
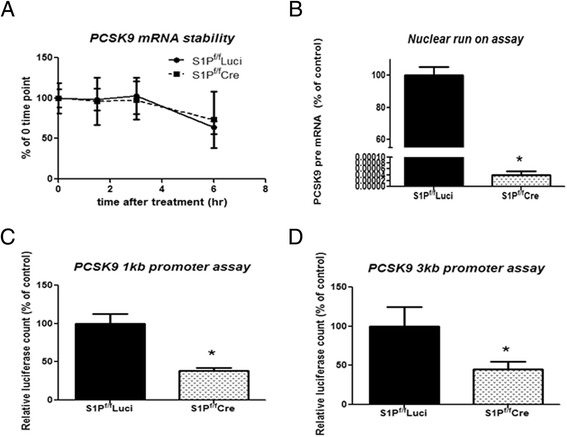
S1P regulates the rate of PCSK9 transcription. **a**, Comparison of PCSK9 mRNA levels at the indicated time points in S1P^f/f^ Luci and S1P^f/f^Cre hepatocytes after treatment with actinomycin D (10 μg/ml). *n* = 6, values plotted as % of the 0 h value; **b**, Comparison of nuclear synthesis of nascent PCSK9 transcripts in S1P^f/f^ Luci and S1P^f/f^Cre hepatocytes using a run-on assay. Values are plotted as the mean of triplicate measurements from one representative experiment. The experiment was repeated three times yielding similar results; **c** and **d**, PCSK9 promoter activities in S1P^f/f^Luci and S1P^f/f^Cre hepatocytes. *N* = 3, each data point represents the mean +/- SD, * denotes *p* < 0.001

To test whether S1P deficiency influenced PCSK9 mRNA stability as opposed to rate of transcription, transcription was blocked using actinomycin D in primary murine hepatocytes from S1P^f/f^Luci and S1P^f/f^Cre mice. As shown in Fig. 5a, the PCSK9 mRNA decay curves of these groups were similar, indicating that hepatic S1P KD did not alter PCSK9 mRNA stability.

To determine whether hepatic S1P deficiency affects the synthesis rate of PCSK9 mRNA, nuclear run-on assays were carried out in primary murine hepatocytes from S1P^f/f^Luci and S1P^f/f^Cre mice. Hepatic S1P KD significantly reduced the amount of newly synthesized PCSK9 pre-mRNA (Fig. 5b), indicating that hepatic S1P controls the rate of PCSK9 mRNA synthesis. Consistent with this result, two murine PCSK9 promoter activities were significantly lower in S1P^f/f^Cre hepatocytes than in control cells (Fig. 5c and d).

### A common pathway accounts for PCSK9 regulation by the SREBPs and S1P

Since S1P is not itself a transcription factor, these results strongly indicate that S1P controls the activity of a transcription factor(s) that influence(s) PCSK9 mRNA synthesis. S1P KD has been reported to block SREBP1 and SREBP2 activation [6]. The downregulation of SREBP1c and SREBP2 mRNA expression levels, and the mRNAS of two of their downstream targets, fatty acid synthase and acetyl CoA carboxylase, were confirmed in S1P^f/f^Cre mice (data not shown).

To determine the role of the SREBPs in regulation of PCSK9 by S1P, SREBP1 and SREBP2 in combination were knocked down using siRNAs in primary S1P^f/f^Luci hepatocytes and S1P^f/f^Cre hepatocytes. Using hepatocytes transfected with a non-targeting scrambled siRNA as controls, efficient KD of SREBP1 and SREBP2 was confirmed in both hepatocyte types. The knockdown efficiency of SREBP1 and SREBP2 was 90–95 % in S1P^f/f^Luci hepatocytes and 55–65 % in S1P^f/f^Cre hepatocytes. Since, S1P^f/f^Cre hepatocytes (siNTC group) already have a reduced expression of SREBP1 and SREBP2 compared to S1P^f/f^Luci hepatocytes (siNTC group) due to reduced processing of SREBPs, we think that the final knockdown of SREBP1 and SREBP2 is equivalent in both control and cre hepatocytes.

**Fig. 6:**
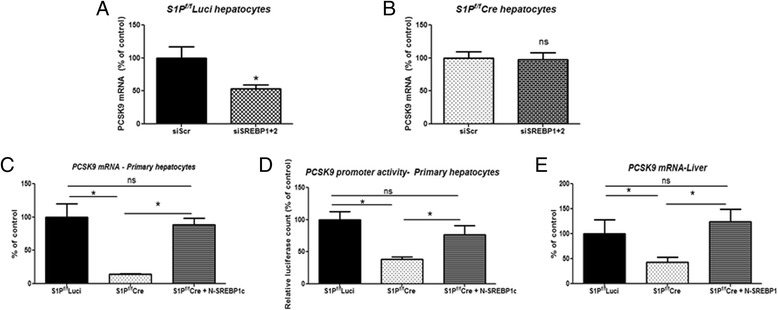
A molecular mechanism for regulation of PCSK9 by hepatic S1P. **a** and **b**, Primary hepatocytes (S1P^f/f^ Luci or S1P^f/f^Cre) were transfected with non-targeting control siRNA (siScr) or SREBP1 + 2 siRNA (siSREBP1 + 2) and cells were harvested 56 h post transfection for RNA isolation. PCSK9 mRNA quantification after combined KD of SREBP1 and SREBP2 in S1P^f/f^ Luci hepatocytes (**a**), and S1P^f/f^Cre hepatocytes (**b**). *N* = 3, each data point represents a mean +/- SD, * denotes *p* < 0.05, ns denotes non significant. All values plotted as % of siScr control; (**c** and **d**), PCSK9 mRNA levels (**c**) and PCSK9 promoter activity (**d**) were measured in S1P^f/f^ Luci and S1P^f/f^Cre hepatocytes after transduction with either Ad-Luci or Ad N-SREBP1c. *N* = 3, each data point represents a mean +/- SD, * denotes *p* < 0.05, ns denotes non significant. All values plotted as % of S1P^f/f^ Luci control; (**e**) Hepatic PCSK9 mRNA levels were measured in three groups of mice: i) S1P^f/f^ Luci (transduced with 10 viral particles of Ad-Luci per mouse); ii) S1P^f/f^ Cre (transduced with 5×10^10^ viral particles of Ad-Cre and 5×10^10^ viral particles of Ad-Luci per mouse); iii) S1P^f/f^Cre + N-SREBP1c (transduced with 5×10^10^ viral particles of Ad-Cre and 5×10^10^ viral particles of Ad-N-SREBP1c per mouse). Mice were fed ad libitum. *N* = 3–4, each data point represents a mean +/- SD, * denotes *p* < 0.05, ns denotes not significant. All values plotted as % of S1P^f/f^ Luci control

Combined KD of SREBP1 and SREBP2 lowered PCSK9 mRNA in S1P^f/f^Luci hepatocytes but not in S1P^f/f^Cre hepatocytes (Fig. 6a and b), suggesting that S1P and the SREBPs contribute to a common mechanism controlling PCSK9 transcription. KD of CREBH or ATF6α in S1P^f/f^Luci hepatocytes did not alter PCSK9 mRNA expression (data not shown), indicating that S1P does not affect PCSK9 expression through CREBH or ATF6α.

### The reduced PCSK9 expression seen in S1P deficiency is restored by complementation of the SREBPs’ function

If lack of activation of the SREBPs’ function is responsible for the reduced PCSK9 mRNA levels seen in S1P-deficient mice, complementation of the SREBPs’ function should restore PCSK9 mRNA levels. To test this, a constitutively active form of SREBP1c, an N-terminal region of murine SREBP1c (N-SREBP1c) [25], was generated.

Hepatocytes from S1P^f/f^Luci and S1P^f/f^Cre mice were transduced with Ad-Luci or Ad-N-SREBP1c. As shown in Fig. 6c, N-SREBP1c overexpression restored PCSK9 mRNA levels in S1P^f/f^Cre cells to the levels seen in S1P^f/f^Luci cells. Similarly, using the same set of hepatocytes, PCSK9 promoter activity was restored in S1P^f/f^Cre cells to the levels seen in S1P^f/f^Luci cells by overexpression of N-SREBP1c (Fig. 6d).

Next, to determine if these results could be recapitulated in vivo, three groups of ad libitum fed mice were studied: (i) S1P^f/f^Luci; (ii) S1P^f/f^Cre; (iii) S1P^f/f^Cre + N-SREBP1c. When N-SREBP1c was overexpressed, mRNAs for both SREBP1c and SREBP2 were elevated (Table 3). As shown in Fig. 6e, the level of PCSK9 mRNA was restored by N-SREBP1c overexpression in the livers of S1P ^f/f^Cre mice (S1P^f/f^Cre + N-SREBP1c) compared with those of S1P^f/f^Cre mice, *p* < 0.05) to an extent similar to that exhibited by the control S1P^f/f^Luci, (S1P^f/f^Cre + N-SREBP1 versus S1P^f/f^Luci, *P* > 0.05). Thus, hepatic S1P KD reduces PCSK9 expression through lack of activation of the SREBPs. As a result of the reduced plasma PCSK9 protein levels, hepatic S1P KD enhances LDLR protein stability and maintains LDLR protein levels despite the reduction in LDLR mRNA expression.

**Table 3:** SREBP1 and SREBP 2 gene expression in livers of mice with SREBP1c-N overexpression

Parameter	S1P^f/f^Luci	S1P^f/f^Cre	S1P^f/f^Cre + N-SREBP1c
Number and sex	4 males	4 males	4 males
SREBP1c mRNA %^#^	100 ± 14	43 ± 9	392 ± 42*
SREBP2 mRNA %^#^	100 ± 27	50 ± 5.3	88 ± 13*

Hepatic mRNA levels of SREBP1c and SREBP2 in the experiment presented in Fig. 6e. Each value represents the mean ± the SD of 4 mice. The statistical significance (Student’s *t* test) between S1P^f/f^Cre and S1P^f/f^Cre + N-SREBP1c was calculated. *P < 0.05 ^#^mRNA expression is expressed as a % of the corresponding S1P^f/f^ Luci value

### Hepatic S1P KD significantly lowers Blp-c levels in LDLR^-/-^ mice

**Fig. 7:**
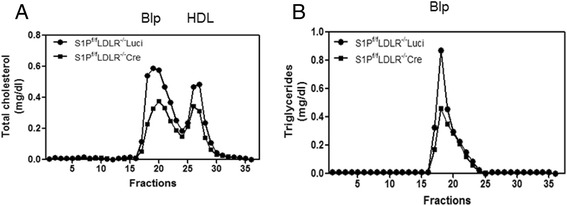
Hepatic S1P KD reduced plasma Blp-c levels in LDLR^-/-^ mice. **a** and **b**, S1P^f/f^LDLR^-/-^ Luci and S1P^f/f^LDLR^-/-^Cre mice. Lipid measurements were carried out on gel filtration fractions from pooled plasma samples of mice in two mouse models of hepatic S1P KD mice. Female mice (*N* = 4, 6–8 weeks of age) were transduced with the indicated adenoviruses. This experiment was repeated three times and similar results were obtained

To investigate whether hepatic S1P KD could reduce the elevated plasma Blp-c levels found in LDLR^-/-^ mice, a line of hepatic S1P KD mice was generated in the LDLR deficient background. The KD of hepatic S1P mRNA was confirmed and was comparable to that in the S1P^f/f^Cre mice (Table 1, line 1, columns 3 and 4). Presence of hepatic S1P^f/f^Cre in the LDLR deficient background resulted in a 39 % and 31 % decrease in TC (*p* < 0.05) and TG (*p* < 0.05) respectively (Table 1, lines 2 and 3, columns 3 and 4). When the blood lipoproteins were analyzed by gel filtration, the relative decreases in both HDL-c and Blp TGs (Fig. 7a and b) were found to be similar to those when hepatic S1P^f/f^Cre was compared with S1P^f/f^Luci (Fig. 1e and f). Significantly, however, hepatic S1P^f/f^Cre in the LDLR deficient background resulted in a significant reduction in plasma Blp-c levels, in terms of both absolute and relative changes (Fig. 7a), compared with the results when hepatic S1P^f/f^Cre was compared with S1P^f/f^Luci (Fig. 1e).

## Discussion

Using multiple mouse models of hepatic S1P deficiency, we have demonstrated a physiological role for hepatic S1P in regulating plasma lipoprotein metabolism through effects on the LDLR and PCSK9. The major findings are:

(i) KD of hepatic S1P (95 % reduction compared with wild type or 75 % reduction compared with S1P^f/f^ mice), resulted in 45 % and 38 % reduction in plasma TC and TG levels, respectively.
(ii) Hepatic S1P deficiency results in discordance between LDLR mRNA and protein expression due to a post-translational stabilization of LDLR protein.
(iii) Hepatic S1P regulates PCSK9 expression through the activation of the SREBPs.
(iv) The lack of normal S1P-PCSK9 regulation of LDLR protein degradation in S1P deficient livers provides an explanation for the discordance in LDLR mRNA and protein expression.
(v) Hepatic S1P deficiency alone has a minimal effect on Blp-c in the S1P^f/f^ background, but produces a significant reduction in LDLR^-/-^mice.

Our study clearly demonstrates a unique role for hepatic S1P deficiency in regulating plasma Blp-c levels. When the LDLR function is intact, hepatic S1P inhibition has little effect on plasma Blp-c levels (Fig. 1d). When the LDLR function is compromised, as occurs in LDLR deficient mice, S1P inhibition potently (~50 %) reduces plasma Blp-c levels (Fig. 7a). Thus, hepatic S1P is a valid target for lowering plasma Blp-c levels in the particular situation where LDLR function is compromised.

While the plasma lipid phenotype associated with hepatic S1P deficiency rather surprisingly is similar to that of transgenic mice overexpressing the nuclear form of SREBP1a in liver (SREBP1a^Tg^ mice) [26], the mechanisms underlying the phenotypes are very different. SREBP1a^Tg^ mice produce large lipid-rich lipoproteins. However, these do not accumulate in plasma because they are cleared through the action of the LDLR whose function is enhanced by up-regulation of SREBP1a. In hepatic S1P KD mice, the plasma Blp-c level, which one would expect to drop as a result of reduced cholesterol synthesis, is maintained due to the impaired function of the LDLR in fasted mice. The doubly mutant SREBP1a^Tg^ LDLR^-/-^ mice exhibit marked increases in plasma Blp-c and triglycerides [26], whereas LDLR^-/-^ S1P^f/f^Cre mice have decreased plasma Blp-c and triglycerides (Fig. 7). These models and their phenotypes highlight the critical role of the LDLR in Blp metabolism in response to either activation or inhibition of the SREBP pathway. Importantly, both activation and inactivation of the SREBP pathway have a minimal impact on plasma Blp-c levels when the LDLR regulation by the SREBPs is intact.

In the work presented here, the maximum reduction of plasma TC and TG that can be achieved through hepatic S1P inhibition is around 50 %. The same is likely true for plasma Blp-c levels. This is very different from inhibition of MTP and apoB, which can produce more profound reductions of plasma Blp-c levels [27, 28]. However hepatic S1P inhibition has a significant advantage over these two approaches, i.e., it is not accompanied by hepatic lipid accumulation. One interesting question is whether hepatic S1P KD has a more profound effect on plasma Blp-c levels in mice on a high fat diet. Hepatic *SREBP cleavage-activating protein (Scap)* gene deletion [29] and Scap protein inhibition by betulin [30] showed that this is indeed the case.

Although SREBPs have been implicated in PCSK9 regulation [13] and S1P controls SREBP activation, we took an unbiased approach to dissect the regulation of PCSK9 by S1P for several reasons: (i), S1P could potentially regulate other transcriptional factors that might also affect PCSK9 mRNA expression; (ii), PCSK9 mRNA is regulated by other coactivators such as hepatic nuclear factor 1α [31, 32]; (iii), PCSK9 protein maturation is influenced by other factors [33]. Under the experimental conditions of this study, we conclude that hepatic S1P mainly regulates plasma PCSK9 levels through controlling SREBPs’ activation.

The maintenance of the endogenous LDLR protein level in S1P deficient mouse livers at the level determined for control mice in the *ad libitum* fed condition (Fig. 2d) can be explained by the increased LDLR protein stability found for the S1P deficient mice, shown in Fig. 3e. Since S1P deficiency reduces plasma PCSK9 levels (Fig. 4d), we believe that the lack of normal S1P-originating production of PCSK9 in such mice is responsible for the discordance in LDLR mRNA (lowered) and protein levels (maintained) seen in the S1P deficient livers (Fig. 2b, d). The S1P-PCSK9 regulation is more prominent in the fed state, which makes sense in terms of the higher level of circulating PCSK9 present in control mice in the fed state (Fig. 4d and [34]). Further studies are required to examine whether lowering of PCSK9 function is solely responsible for the effect on LDLR protein in the S1P deficient mice.

Our results also reveal a complex role for hepatic S1P in regulation of LDLR function and plasma Blp-c levels. On the one hand, hepatic S1P KD decreases LDLR mRNA expression through reducing the SREBPs, and on the other hand, increases LDLR protein levels through down-regulation of PCSK9 expression, again, mediated through the SREBPs. The net effect of hepatic S1P KD on LDLR level/function is unfavourable under fasting conditions (Fig. 2f). A similar type of regulation likely exists for plasma Blp-c metabolism. Hepatic S1P KD is known to decrease cholesterol biosynthesis which would likely decrease VLDL secretion, at the same time reducing VLDL/LDL clearance through lowered levels of the LDLR. Thus the net effect of hepatic S1P KD on plasma Blp-c is minimal. It is possible that these results may also be obtained using inhibitors of the SREBP pathway other than S1P KD.

To differentiate the effect of S1P on LDLR mRNA from its effect on LDLR protein stability, we developed a novel and sensitive *in vivo* LDLR reporter assay to measure LDLR protein turnover. The assay, which is modeled on the conventional dual reporter assay for promoter analysis, is feasible, because fusion of Gaussia luciferase with the LDLR did not affect LDLR location and function. In light of the recent discovery of novel modulators of LDLR protein turnover, such as IDOL, Annexin A2, ARH and Rab5 [20, 22, 35, 36], we envision that this LDLR protein reporter assay could have broad applications. With some modifications, the assay could be used to determine the kinetics of LDLR protein turnover since it can differentiate endogenous from exogenous LDLR. This reporter system would also permit identification and validation of novel post-translational modulators of LDLR *in vivo*.

Inhibition of hepatic S1P has been shown to reduce plasma TC and TG levels [6, 8]. Apart from the known decreased lipid *de novo* synthesis, it was not apparent that there was an additional mechanism(s) by which S1P inhibition lowered plasma lipids. Here we show that hepatic S1P deficiency reduces hepatic PCSK9 expression and thus degradation of the LDLR. Because PCSK9 has been shown to be involved in VLDL secretion [37], further studies are required to determine whether hepatic S1P KD also reduces VLDL secretion through inhibition of PCSK9.

## Conclusions

**Fig. 8:**
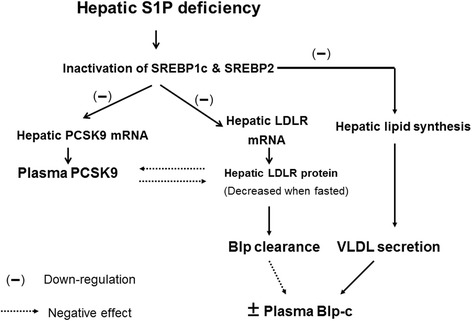
A working model of S1P regulation of plasma Blp-c levels

In summary, our studies clearly demonstrate the physiological role of hepatic S1P deficiency in PCSK9 and LDLR regulation under various conditions. Hepatic S1P deficiency represses activation of the SREBP proteins. A reduced number of activated SREBPs then bind the LDLR and PCSK9 gene promoters, resulting in less transcription of their mRNAs and reduced plasma PCSK9 protein levels. The reduction of PCSK9 mRNA and protein is more prominent in the fed state, which likely contributes to the increased LDLR protein stability and the maintenance of hepatic LDLR protein levels despite the reduced LDLR mRNA. In the fasting state, hepatic S1P KD has less effect on plasma PCSK9 concentrations and as a result, liver LDLR protein levels are lower (Fig. 8). These regulatory processes are critical for fine-tuning both LDLR protein at the post-translational level and plasma Blp-c levels.

## Abbreviations

Blp: apoB-containing lipoprotein
KD: Knockdown
Ad: Adenovirus
S1P: Site-1 protease
PCSK9: Proprotein convertase subtilisin kexin type 9
Ab: Antibody
SREBP: Sterol regulatory element binding protein
LDLR: Low density lipoprotein receptor
